# Case Report: From IgA nephropathy to pneumonia, periappendiceal abscess, pulmonary arterial hypertension, and basal ganglia calcification: a case of chronic active Epstein-Barr infection in a child

**DOI:** 10.3389/fped.2025.1589151

**Published:** 2025-08-04

**Authors:** Shuying Fan, Xin Wang, Ning Wei, Qiumei Zhou, Wenhong Wang

**Affiliations:** Department of Nephrology, Tianjin Children's Hospital, Tianjin Key Laboratory of Birth Defects for Prevention and Treatment, Tianjin, China

**Keywords:** chronic active Epstein–Barr infection, IgA nephropathy, pneumonia, enteritis, pulmonary arterial hypertension, child

## Abstract

Chronic active Epstein–Barr virus infection (CAEBV) is a lymphoproliferative disorder characterized by persistent EBV infection, which can lead to multi-organ involvement. This case describes a child with CAEBV who initially presented with IgA nephropathy (IgAN) without characteristic infectious mononucleosis (IM) features. The patient initially presented with intermittent gross hematuria, Renal biopsy confirmed focal proliferative IgAN, and the child was treated with methylprednisolone pulse therapy followed by oral prednisone. During follow-up, the patient sequentially developed pneumonia caused by co-infection with EBV and *Staphylococcus aureus*, periappendiceal abscess, pancytopenia, Intermittently elevated peripheral blood EBV-DNA load, raising suspicion of CAEBV. Further investigations revealed the following findings: echocardiography demonstrated pulmonary arterial hypertension (PAH); cranial CT showed multiple bilateral basal ganglia calcifications; bone marrow biopsy detected EBV-DNA positivity (6.5 × 10³ copies/ml); renal tissue immunohistochemistry showed CD8+ cells (scattered, −50/HPF) and CD4+ cells (focal, −40/HPF), with negative LMP-1 but scattered EBER+ signals (−25/HPF). Based on persistently elevated peripheral blood EBV-DNA load, EBER-positive lymphocyte infiltration in renal tissue, evidence of multi-organ involvement, the diagnosis of CAEBV was established.

## Introduction

CAEBV is a lymphoproliferative disorder characterized by clonal proliferation of EBV-specifically targeted lymphocytes. It is characterized by recurrent or prolonged IM-like symptoms with elevated peripheral blood EBV levels in patients without known immunodeficiency, malignancy, or autoimmune disease. The main pathophysiologic feature is persistent EBV infection of T cells, NK cells, or B cells with clonal proliferation and infiltration of multiple organs, leading to fatal complications. The epidemiological features of the significantly high prevalence in East Asian populations suggest that genetic polymorphisms in host immune-related genes may play a key role in the pathogenesis (1, 2). The clinical heterogeneity of this disease is particularly striking, with approximately 50% of cases presenting with the classic IM-like triad (fever, enlarged lymph nodes, hepatosplenomegaly), while the remainder of patients present with non-specific symptoms of multi-system involvement. Cutaneous mucosal manifestations (anaphylactic reactions to mosquitoes, bullous pemphigoid), gastrointestinal symptoms (intractable diarrhea), and ocular lesions (uveitis) are relatively common atypical presentations. Disease progression may follow a biphasic pattern: while some patients remain clinically stable for extended periods, approximately 60% of cases advance to fatal complications within 5–10 years. The most common complications include hemophagocytic lymphohistiocytosis (21%), coronary artery aneurysms (21%), hepatic failure (18%), basal ganglia calcification (18%), malignant lymphoma (16%), and interstitial pneumonitis (12%) (3). Notably, simultaneous multi-organ involvement is exceedingly rare, and renal lesions as the initial disease manifestation have not been reported in the existing literature.

This article reports a pediatric case of CAEBV with a distinctive clinical trajectory: the child initially presented with IgAN and subsequently developed multi-system involvement including hematologic abnormalities (anemia, thrombocytopenia), severe pneumonia (EBV-associated), periappendiceal abscess (simulated purulent appendicitis), PAH and neurologic damage. The clinical distinctiveness of this case is manifested in: (1). It provides a breakthrough demonstration of the intrinsic link between IgAN and EBV infection (2). The gastrointestinal complication manifested as a rare surgical acute abdomen-like phenotype. (3). The PAH development preceded the clinical manifestations of cardiopulmonary decompensation. This report aims to alert clinicians that pediatric CAEBV can present as a “great masquerader”, when multi-organ involvement defies explanation by a single disease entity, a high index of suspicion for EBV-associated disorders is warranted to prevent treatment delays resulting from missed diagnosis.

## Case description

### First admission (2020.12): initial diagnosis of IgAN

**Figure 1:**
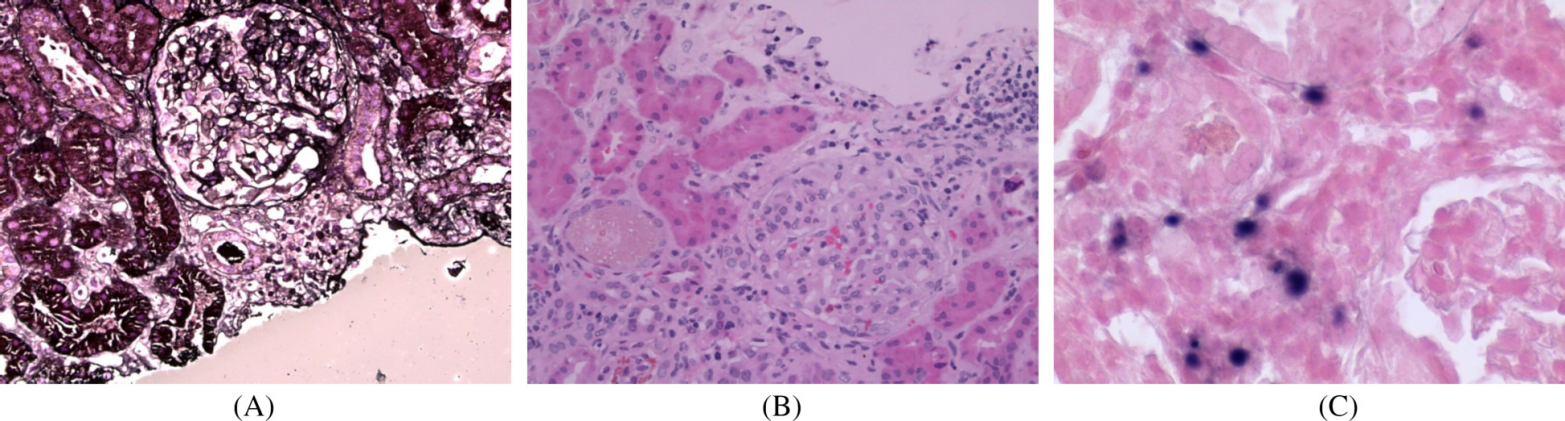
Renal pathological examination: **(A)** periodic acidsilver metheramine (PASM) staining (×400); **(B)** H&E staining (×400); **(C)** H&E staining (×400). **(A,B)** Light microscopic examination demonstrated one small cellular fibrotic crescent, vacuolar and granular degeneration of renal tubules, focal severe vacuolization, and focal lymphocytic and mononuclear cell infiltration in the renal interstitium. **(C)** *in situ* hybridization demonstrated scattered EBER-positive cells (approximately 25/HPF).

A 9-year-old girl presented with intermittent gross hematuria for 2 years. She denied any history of exposure to infectious diseases or family history of kidney disease and genetic disorders. Physical examination revealed hypotension (85/50 mmHg), normotrophic appearance, no edema or palpable superficial lymphadenopathy, and splenomegaly (spleen palpable at costal margin). Laboratory tests revealed hemoglobin 81 g/L, leukopenia (WBC 2.0 × 10⁹/L), thrombocytopenia (platelets 133 × 10⁹/L), reticulocyte 2.21%, elevated C-reactive protein (CRP 14.3 mg/L), erythrocyte sedimentation rate (ESR) increased to 74 mm/h, and a negative Coombs test. Blood chemistries showed creatinine 73 μmol/L and urea nitrogen 6.74 mmol/L. Urinalysis demonstrated proteinuria (1+), hematuria (4+ RBCs), and elevated 24 h urinary protein (UPro-24 h) excretion (347.5 mg/24 h). Tests for immunoglobulin indicated IgA was significantly elevated (5.71 g/L), complement 3 (C3 0.82 g/L) was decreased, anti-nuclear antibody (ANA), extractable nuclear antigen (ENA), anti-neutrophil cytoplasmic antibody (ANCA) were negative. Virological studies detected low-level EBV viremia (1.1 × 10³ copies/ml) with positive EBNA-IgG, and excluded hepatitis virus coinfection. The detailed laboratory findings are shown in Table 1. Renal ultrasound demonstrated diffuse renal parenchymal lesions, Thoracoabdominal CT revealed bilateral upper lobe inflammatory consolidations with hepatosplenomegaly. Renal pathology (Figures 1A,B) demonstrated: (1) Light microscopy—25 glomeruli with 1 fibrocellular crescent, diffuse mesangial hypercellularity, and focal endocapillary proliferation; (2) Immunofluorescence—mesangial IgA++, C3++, IgM+, and fibronectin+ deposits; (3) Electron microscopy—mesangial electron-dense deposits and partial foot process effacement. The patient was diagnosed with focal proliferative IgA nephropathy, acute kidney injury (KDIGO stage 1), EBV viremia, and moderate microcytic anemia. Methylprednisolone pulse therapy was administered, followed by oral prednisone, captopril and dipyridamole, which resulted in remission of gross hematuria, and outpatient follow-up showed remission of proteinuria (PRO-), but persistence of microscopic hematuria (RBC 2+).

**Table 1:** Laboratory findings from four hospital admissions.

Clinical variables	First hospitalization	Second hospitalization	Third hospitalization	Fourth hospitalization
Blood routine				
Hb, g/L (118–156)	81	94	94	123
WBC count, 10^9^/L (4.3–11.3)	2.0	3.63	14.35	2.12
PLT count, 10^9^/L (167–453)	133	153	224	113
Urine routine				
PRO (Negative)	+	Negative	Negative	3+
RBC,/HP (0–3)	4+	+	1–3	4+
UPro-24 h, mg/24 h (0–150)	347.5	173.6		473.5
Serum				
Urea, mmol/L (1.78–6.42)	6.74	5.77	4.43	10
Creatinine, μmol/L (29–56)	73	28	36	73
ALT, U/L (7–40)	24	39	10	18
AST, U/L(13–35)	51	22	28	39
Inflammatory index				
CRP, mg/L (0–8)	14.3	153	134	189.8
ESR, mm/h (0–20)	74	67		17
Immunological indicators				
ANA profile (Negative)	Negative	Negative		Negative
ANCA profile (Negative)	Negative	Negative		Negative
IgG, g/L (5.72–14.74)	13.19	7.65	8.3	11.64
Ig A, g/L (0.34–3.05)	5.71	3.7	4.49	5.15
IgM, g/L (0.31–2.08)	0.53	0.63	0.93	0.51
C3, g/L (0.9–1.8)	0.82	1.45g	1.25	1.33
C4, g/L (0.1–0.4)	0.21	0.48	0.31	0.41
Peripheral blood EBV				
EBV-DNA, copies/ml (<10^3^)	1.1 × 10^3^	9.4 × 10^4^		6.7 × 10^3^
EBVCA-IgM, AU/ml (0–3)	Negative	Negative		Negative
EBNA-IgG, AU/ml (0–2)	10.55	10.55		5.06
BALF EBV				
EBV-DNA		7.2 × 10^6^		
mNGS EBV reads		4,113		
Bone Marrow				
EBV-DNA, copies/L				6.5 × 10^3^

ALT, alanine laminotransferase; AST, aspartate aminotransferase; C4, complement 4.

### Second admission (2021.02): EBV-associated pneumonia

**Figure 2:**
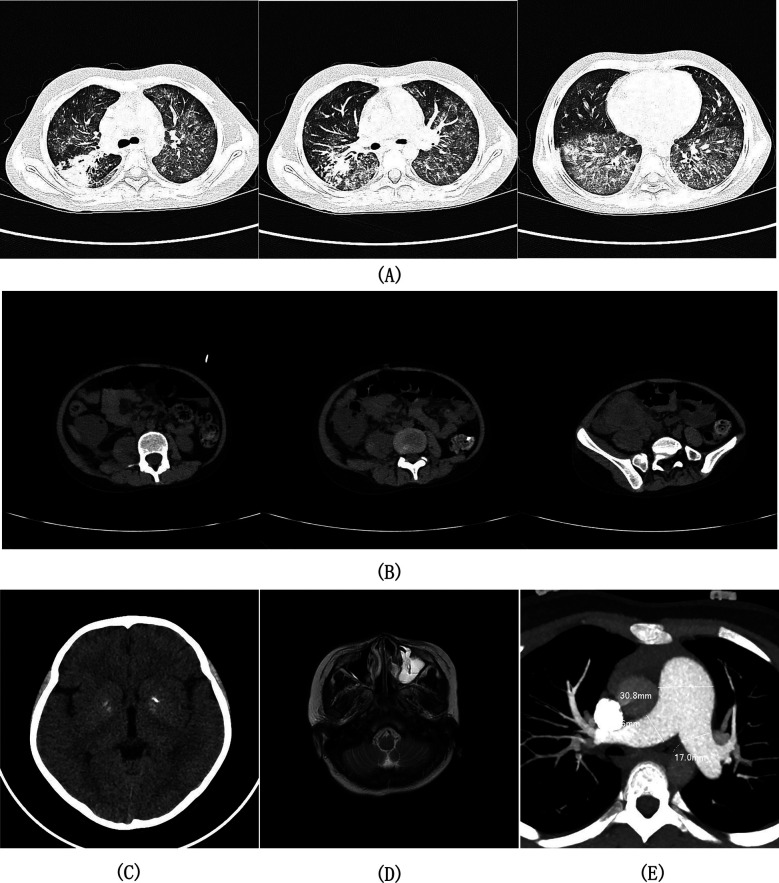
Imaging findings from axial computed tomography (CT) of the chest, abdominopelvic region, and cranial cavity; axial magnetic resonance imaging (MRI) of the head; and contrast-enhanced CT angiography (CTA) of the thoracic arteries. **(A)** Chest CT demonstrated bilaterally increased pulmonary markings with heterogeneous attenuation, demonstrating scattered patchy consolidations and ground-glass opacities throughout both lung fields, along with small subpleural nodules in the left lung and right lower lobe. **(B)** Abdominopelvic CT demonstrated focal bowel wall thickening with heterogeneous soft-tissue density mass formation in the right lower abdomen, right psoas muscle swelling, enlarged root-of-mesentery lymph nodes, and pelvic free fluid. **(C)** Cranial CT demonstrated symmetrical calcific foci in the bilateral basal ganglia regions. **(D)** T2-weighted head MRI demonstrated left maxillary sinus mucosal thickening with inflammatory changes. **(E)** CTA of the thoracic arteries demonstrated dilatation of the main pulmonary artery and bilateral pulmonary arteries.

Two months after her initial discharge, she was readmitted to the hospital with “fatigue for 9 days, diarrhea with fever for 3 days, and cough for 1 day”. Physical examination revealed early signs of shock: blood pressure 80/50 mmHg, pulse 115 beats/min, breathing 30 times/min, and wet rales in the right lung. Laboratory and imaging revealed significantly elevated CRP (153 mg/L), EBV-DNA load surge (9.4 × 10^4^ copies/ml). Bronchoalveolar lavage fluid (BALF) metagenomic next-generation sequencing (*mNGS)* detected: *Staphylococcus aureus* (87 reads) and EBV (4,113 reads). Chest CT showed diffuse patchy consolidations and ground-glass opacities in both lungs, small subpleural nodules in the left lung and lower lobe of the right lung, bilateral pleural thickening, and increased texture with uneven transparencyin both lungs (Figure 2A). The patient was diagnosed with severe pneumonia (EBV and *Staphylococcus aureus* coinfection) and septic shock. The patient received anti-shock therapy, prednisone (45 mg/day), anti-infective treatment with linezolid combined with acyclovir, and intravenous immunoglobulin (IVIG) support, demonstrating clinical improvement after 8 days of hospitalization followed by gradual prednisone tapering.

### Third admission (2022.01): periappendiceal abscess

She was admitted to the hospital with “metastatic right lower abdominal pain for 10 days”, Physical examination revealed signs of peritoneal irritation in the right lower quadrant, including tenderness, rebound tenderness, and guarding. Laboratory tests and imaging showed Hb 94 g/L, WBC 14.35 × 10^9^/L, CRP 109.45 mg/L. Abdominal CT indicated focal bowel wall thickening with an irregular soft-tissue density mass and enlarged peri-mesenteric lymph nodes in the right lower abdomen, suggestive of a periappendiceal abscess (Figure 2B). She was given fasting, rehydration, and ertapenem combined with ornidazole for anti-infection, and was discharged after the symptoms resolved.

### Fourth admission (2023.02): confirmed diagnosis of CAEBV and multisystem damage

She was admitted to the hospital with “headache and gross hematuria for 3 days”. Physical examination revealed clear consciousness, no positive neurological signs, and an accentuated second heart sound (P₂). EBV-associated laboratory findings revealed: serum EBV DNA persistently positive (6.7 × 10^3 ^copies/ml), bone marrow EBV DNA 6.5 × 10^3^ copies/ml, lymphocyte subset test for EBV infection showed: CD4^+^T cells: 3.4 × 10^4^ per million cells, CD8^+^ T cells: 3.3 × 10^5^ per million cells, B cells: 1.25 × 10^4^ per million cells, NK/NKT cells: 2.3 × 10^4^ per million cells. Supplementary immunohistochemical and EBER *in situ* hybridization testing of the 2020 renal tissue specimen revealed: scattered CD8+ T-cell infiltration (approximately 50 cells/HPF); focal CD4+ T-cell clusters (locally approximately 40 cells/HPF); scattered EBER-positive signals (approximately 25 cells/HPF); and negative EBV latent membrane protein 1 (LMP-1) expression. Multi-system evaluation revealed: (1) Neurological—Cranial CT (Figure 2C) showed bilateral basal ganglia calcifications; (2) Paranasal sinuses—MRI demonstrated bilateral mucosal thickening and inflammation in the maxillary, ethmoid, sphenoid, and frontal sinuses (Figure 2D); (3) Cardiovascular—Echocardiography showed moderate PAH (PG: 51 mmHg, estimated pulmonary artery systolic pressure −56 mmHg), with CT angiography of thoracic arteries revealing dilatation of the main pulmonary artery and bilateral pulmonary arteries (Figure 2E). Genetics: Whole-exome sequencing identified no pathogenic single-nucleotide variants or insertion-deletion variants correlating with the clinical phenotype. Final diagnosis: CAEBV, EBV-associated IgAN, PAH, basal ganglia calcification. The patient was treated with ganciclovir antiviral therapy and an increased prednisone dosage (30 mg/day), which alleviated the headache and resolved gross hematuria. Hematopoietic stem cell transplantation (HSCT) was recommended as the next therapeutic step. Following discharge, the patient continued follow-up treatment at an external institution. Telephone follow-up revealed disease progression to lymphoma 6 months later, culminating in mortality at the 1-year mark.

## Discussion

As a rare systemic lymphoproliferative disease, CAEBV is challenging to diagnose due to its multi-organ involvement. By integrating the literature review and case analysis, this study systematically explored the mechanism of CAEBV-associated renal injury for the first time and revealed the clinical features of rare complications in the digestive tract and cardiovascular system, which provided a new chain of evidence to improve the diagnostic and therapeutic pathway of this disease.

### CAEBV and renal complications

Although tubulointerstitial nephritis and nephrotic syndrome have been reported as renal complications of EBV infection, the specific mechanisms by which EBV infection leads to renal injury have not been fully elucidated. Some studies suggest that EBV may induce renal pathology through direct viral invasion or immune-mediated mechanisms. For example, some patients had positive EBER *in situ* hybridization tests in renal tissues, suggesting that there may be direct viral damage to renal tissues (4). Another retrospective study of a large sample of renal puncture cases found that EBV DNA was detected in renal tissues of patients with IgA nephropathy, membranous nephropathy, and focal segmental glomerulosclerosis, and was particularly common in glomerular diseases with immune complex deposition in the mesangial area, suggesting that EBV may damage the glomerular mesangium through an immunoglobulin-mediated mechanism (5).

However, CAEBV-associated renal complications are clinically rare, and systematic studies of their relationship between CAEBV and renal pathology are still lacking. Some case reports have been published suggest possible pathological mechanisms, for example, a 70-year-old CAEBV patient in Japan developed acute tubulointerstitial nephritis and minimal change nephrotic syndrome, and renal biopsy showed EBV-positive lymphocytic infiltration, and the patient's renal function improved spontaneously without specific treatment (6). Another case of a 54-year-old female patient presented with prolonged fever and progressive deterioration of renal function, and renal biopsy showed diffuse small-to-medium lymphocyte infiltration in the tubulointerstitium, and immunohistochemistry showed a massive infiltration of CD4+ T cells and distribution of EBER-positive cells among CD4+ T cells (7). These cases suggest that EBV may cause renal injury through direct lymphocyte infiltration. Notably, reports of CAEBV complicated by IgA nephropathy are extremely rare. Sato et al. (8) described an elderly CAEBV patient whose renal biopsy showed features of IgAN, but no EBV genome was detected in the renal tissue. The patient exhibited chronic EBV infection in B cells, leading the authors to hypothesize that EBV might induce pathogenic IgA production via hypergammaglobulinemia, triggering glomerular injury through antibody-mediated humoral immune mechanisms. In the present case, the detection of EBER-positive cells in renal tissues suggests that EBV may be involved in renal injury through direct renal tissue infiltration, and in addition, the presence of EBV-infected B and T cells in the peripheral blood suggests that antibody-mediated humoral immune mechanisms may play an important role in disease progression. Importantly, multiple mechanisms may coexist in the same patient, which poses a new challenge for the selection of targeted therapeutic strategies.

### Gastrointestinal involvement: a diagnostic pitfall

Gastrointestinal manifestations of CAEBV are uncommon but clinically significant. These cases often present with diarrhea and predominantly involve the colon, potentially mimicking infectious gastroenteritis or inflammatory bowel disease in initial presentation (9, 10). Systemic CAEBV usually presents with fever, lymphadenopathy and splenomegaly, while gastrointestinal involvement may lead to abdominal pain and diarrhea. In contrast, primary gastrointestinal CAEBV usually presents with chronic recurrent gastrointestinal symptoms accompanied by intermittent high fever. When patients do not present with typical IM-like symptoms as their initial manifestation, they are often misdiagnosed with other conditions, leading to delayed treatment. Chen et al. reported a case of a 33-year-old male patient with intermittent fever, abdominal pain and diarrhea for over 3 years and peripheral blood EBV DNA test results of 9.95 × 10^6^ copies/L. Enteroscopy showed multiple deep ulcers in the ileocecal valve and throughout the colon, with ring ulcers being the most prominent, and symptoms improved after symptomatic treatment, but abdominal pain worsened with dark stools and he was readmitted to hospital. An enhanced CT of the lower abdomen showed inhomogeneous thickening of the cecum and terminal ileum with multiple enlarged lymph node shadows in the peripheral space, suggesting inflammatory bowel disease and appendiceal abscess. Partial ileal resection and terminal ileostomy were performed under general anesthesia. Pathological examination of the resected bowel revealed diffuse EBER-positive cells (>90%) in the lesions, confirming gastrointestinal CAEBV (11). This child was admitted to hospital with metastatic right lower abdominal pain and imaging suggestive of a periappendiceal abscess, which resolved with supportive treatment. Although EBER *in situ* hybridization of intestinal tissue was not performed, subsequent confirmation of CAEBV diagnosis and imaging findings suggested gastrointestinal involvement as a likely manifestation of CAEBV. These cases highlight that gastrointestinal symptoms accompanied by persistent EBV emia, even in the absence of classic IM-like features, should prompt consideration of CAEBV in the differential diagnosis. Timely histopathological evaluation is critical for definitive diagnosis. For patients meeting CAEBV diagnostic criteria with concurrent gastrointestinal symptoms, endoscopic evidence of intestinal lesions, and histopathological confirmation of EBER-positive cells in affected tissues, a diagnosis of CAEBV-associated gastrointestinal involvement can be established.

### Pulmonary arterial hypertension: an rare cardiovascular complication

CAEBV is a clinically heterogeneous disorder characterised by multiorgan involvement, including cardiovascular manifestations. Epidemiological data indicate circulatory system complications occur in 17.9% of CAEBV cases (12), predominantly presenting as coronary artery aneurysms or myocarditis (13), CAEBV-associated PAH remains relatively rare. Fukuda et al. (14) reported an 11-year-old boy whose initial manifestation was isolated PAH, lacking typical major symptoms associated with CAEBV such as fever, lymphadenopathy, or splenomegaly, with only persistent mild liver dysfunction observed. After 24 months of follow-up, the patient developed worsening liver function, hepatosplenomegaly, coronary artery aneurysms, and EB viremia, leading to the definitive diagnosis of CAEBV. Ba et al. (15) described an 8-year-old boy who presented with recurrent lower leg ulcers persisting for over 3 years. Histopathological examination of the skin biopsy samples revealed vasculitis, and *in situ* hybridization testing was positive for EBER, elevated plasma EBV load was detected, and echocardiography showed aortic sinus dilation accompanied by PAH (PASP = 54 mmHg). The patient had no PAH-related symptoms, the family declined further investigations. The child was treated with low-dose prednisone. One year later, he returned with edema and easy fatigue during walking. In addition to PAH, systemic vasculitis was identified, and a diagnosis of CAEBV was established. His symptoms improved after treatment with immunosuppressants and PAH-targeted medications. In the present case, physical examination revealed a hyperactive second heart sound in the area of the pulmonary valve, and further evaluation of cardiovascular showed moderate PAH, but no common cardiovascular complications such as myocarditis or coronary artery aneurysm. This case demonstrates that the onset of PAH may precede the clinical manifestations of cardiopulmonary decompensation. EBV-infected cells can cause vasculitis and finally PAH (16). We recommend regular cardiovascular function assessments for all CAEBV patients, regardless of the presence of exertional dyspnea or cyanosis, to enable early detection and intervention for severe complications such as PAH. Additionally, CAEBV should be included in the differential diagnosis of secondary PAH, with EBV DNA testing as a mandatory screening to avoid missed diagnoses.

#### Neurological complications of CAEBV

Children with CAEBV often develop various central nervous system (CNS) complications during the course of the disease. Studies indicate that 7%–23.9% of CAEBV patients may experience CNS involvement, with clinical manifestations including meningitis, encephalitis, encephalomyelitis, and basal ganglia calcification (3, 17–18). The first autopsy-confirmed case of severe and extensive CNS involvement in a CAEBV patient was reported in a 27-year-old Japanese male. This patient primarily exhibited recurrent respiratory and CNS symptoms, ultimately progressing to hemophagocytic lymphohistiocytosis. Imaging studies revealed multiple nodular lesions in bilateral lungs and abnormal signal areas in the brain and spinal cord. EBV-DNA was detected in the cerebrospinal fluid. Autopsy findings confirmed diffuse infiltration of lymphocytes and/or macrophages in multiple organs, including the lungs, liver, kidneys, spleen, myocardium, bone marrow, and CNS, with the most severe involvement observed in the spinal cord and brainstem. Immunohistochemistry showed EBER positivity in CD3+ cells in the brainstem (19). The clinical manifestations, laboratory findings, and neuroimaging features of CNS complications in CAEBV are often nonspecific. Literature reports indicate that 18% of CAEBV patients exhibit basal ganglia calcification (3), which can lead to various neurological symptoms, such as cognitive decline, movement disorders (ataxia, dystonia, Parkinsonism), neuropsychiatric symptoms (depression, anxiety, psychosis) and various other signs (migraine, speech disorders, pain, seizures) (20). In this case, the child presented only with headache and no other neurological symptoms or signs. This highlights the importance for clinicians to systematically screen for neurological complications in pediatric CAEBV patients.

### Challenges in the diagnosis and treatment of CAEBV

CAEBV with multi-organ involvement are exceedingly rare. Vial et al. documented a representative case of T-cell type CAEBV presenting with oral ulcerations, chronic sinusitis, pneumonia, pleural/pericardial effusion, hepatic impairment, ocular myositis, and tubulointerstitial nephritis. Histopathological analyses of multiple biopsy specimens (oral mucosa, liver, kidney, and skin) consistently demonstrated EBV-positive lymphocytic infiltration. Although combination therapies including corticosteroids, rituximab, cyclosporine, interferon *α*-2a, and CHOP chemotherapy regimen transiently ameliorated clinical symptoms and reduced peripheral EBV-DNA viral load, the patient ultimately progressed to multi-organ failure with concomitant gastrointestinal hemorrhage leading to mortality (21). To our knowledge, no published cases have reported IgA nephropathy as the initial presentation of systemic CAEBV involvement.

CAEBV presents with highly heterogeneous and nonspecific clinical manifestations, frequently resulting in delayed diagnosis. The 28-month diagnostic process in this case not only demonstrated the diagnostic challenges of CAEBV but also reveals significant knowledge gaps in current clinical practice. In 2022, the Ministry of Health Labor and Welfare research team in Japan revised the diagnostic criteria for CAEBV, requiring: (1) persistent or recurrent infectious mononucleosis-like symptoms for >3 months; (2) Detection of an increased number of EBV genomes in peripheral blood and/or affected tissues; (3) Detection of EBV-infected T or NK cells in peripheral blood and/or affected tissues; and (4) Chronic illness that cannot be explained by other known disease processes at the time of diagnosis. All four criteria should be met before CAEBV can be diagnosed. Infectious mononucleosis-like symptoms include fever, swollen lymph nodes, and hepatosplenomegaly. Additional complications include hematological, digestive tract, neurological, pulmonary, ocular, dermal (hydroa vacciniforme lymphoproliferative disorder and severe mosquito bite allergy), and cardiovascular (aneurysm and valvular disease) disorders (22). For pediatric patients with atypical presentations but multi-organ involvement (particularly renal, gastrointestinal, cardiovascular, or neurological systems), CAEBV should be considered in the differential diagnosis. The diagnosis should be confirmed through detection of peripheral blood EBV-DNA load, and/or identification of EBER-positive cells in affected tissues combined with immunophenotyping to characterize EBV-infected lymphocyte subsets.

Since CAEBV is fundamentally characterized by EBV-infected T/NK cell proliferation, precise identification of the EBV-infected lymphocyte subsets is crucial for both definitive diagnosis and differentiation from other EBV-associated disorders. Epidemiological data from Asian populations indicated that approximately 60% of CAEBV cases involved T-cell lineage, with CD4+ predominance over CD8+ T cells (23). Coinfection of T cells and NK cells has been documented in some patients (24). Prognostic analysis reveals significantly inferior survival rates in T-cell-type CAEBV compared to NK-cell-type (5-year survival rates: 0.59 vs. 0.87) (25). In a cohort study of 112 pediatric CAEBV cases conducted by Wei et al., CD8+ T-cell predominance was associated with the worst clinical outcomes among all subtypes (including CD4+ and CD56+) and was established as an independent predictor of poor prognosis (26). Lymphocyte immunophenotyping in our patient demonstrated multi-lineage EBV infection (CD4+ T cells, CD8+ T cells, NK/NKT cells, and B cells), with CD8+ T-cell dominance corresponding to the observed unfavorable clinical course.

Currently, there is no standardized treatment protocol for chronic active EBV infection (CAEBV). Although antiviral medications, immunosuppressive therapy, and chemotherapy can temporarily control disease activity, most patients eventually experience relapse. In this case, the pediatric patient initially responded to glucocorticoid and antiviral therapy with symptomatic improvement, but subsequently developed disease recurrence. Allogeneic hematopoietic stem cell transplantation (HSCT), first successfully implemented as a curative treatment for CAEBV in 2000 (27), has been incorporated into clinical guidelines. However, treatment selection requires careful evaluation because some CAEBV cases may follow a self-limiting course requiring only supportive care, while HSCT carries substantial risks of treatment-related complications. Current evidence does not support routine HSCT for all CAEBV patients, necessitating individualized treatment strategies based on comprehensive clinical assessment.

## Data Availability

The original contributions presented in the study are included in the article/Supplementary Material, further inquiries can be directed to the corresponding author.
